# Innovative Nanocarriers: Elastic Aspasomes Loaded with Metformin Hydrochloride for Effective Management of Melasma: In Vitro Studies and Clinical Appraisal

**DOI:** 10.3390/pharmaceutics18030364

**Published:** 2026-03-14

**Authors:** Rofida Albash, Abdurrahman M. Fahmy, Maha H. Ragaie, Shimaa S. Ahmed, Rabab A. El-Gazar, Amira B. Kassem, Manar Adel Abdelbari, Hoda A. Salem, Asmaa Saleh, Shaimaa Mosallam

**Affiliations:** 1 Department of Pharmaceutics, College of Pharmaceutical Sciences and Drug Manufacturing, Misr University for Science and Technology, Giza 12568, Egypt; 2 Department of Pharmaceutics and Industrial Pharmacy, Faculty of Pharmacy, Cairo University, Cairo 12613, Egypt; abdurrahaman.fahmy@pharma.cu.edu.eg; 3 School of Life and Medical Sciences, University of Hertfordshire Hosted by Global Academic Foundation, New Administrative Capital, Cairo 11865, Egypt; 4 Department of Dermatology, STD’s and Andrology, Faculty of Medicine, Minia University, Al-Minya 61111, Egypt; mahahussien333@yahoo.com (M.H.R.);; 5 Department of Clinical Pharmacy, October 6 University, Giza 12585, Egypt; rababahmed@o6u.edu.eg; 6 Clinical Pharmacy and Pharmacy Practice Department, Faculty of Pharmacy, Damanhour University, Damanhour 22514, Egypt; amira.kassem@pharm.dmu.edu.eg; 7 Department of Pharmaceutics and Industrial Pharmacy, Faculty of Pharmacy, October 6 University, Giza 12585, Egypt; manaradel.ph@o6u.edu.eg (M.A.A.); shaimaamosallam@o6u.edu.eg (S.M.); 8 Pharmacy Practice Department, University of Tabuk, Tabuk 71491, Saudi Arabia; hsalem@ut.edu.sa; 9 Department of Pharmaceutical Sciences, College of Pharmacy, Princess Nourah Bint Abdulrahman University, Riyadh 11671, Saudi Arabia; asali@pnu.edu.sa

**Keywords:** melasma, metformin hydrochloride, aspasomes, clinical study, topical drug delivery

## Abstract

**Background/Objectives**: Aspasomes (ASPs) are composed of ascorbyl palmitate (AP), which has antioxidant activity. The objective of this study was the formulation of aspasomes (ASPs) loaded with metformin hydrochloride (MFC) for the topical treatment of melasma. **Methods**: MFC-ASPs were prepared using the thin-film method with different amounts of phospholipid and ascorbyl palmitate (AP) in the absence or presence of ethanol surfactant. The prepared formulations were optimized using a D-optimal mixture. The assessed responses included entrapment efficiency (%EE), particle size (PS), polydispersity index (PDI), and zeta potential (ZP). **Results**: The optimum OASPs, composed of 193.121 mg PC and 30 mg AP, exhibited spherical vesicles with an EE% of 87.50 ± 0.33%, PS of 264.47 ± 0.02 nm, PDI of 0.423 ± 0.001, and ZP of −21.67 ± 0.12 mV. The optimum formula represented a spherical morphology using transmission electron microscopy, along with sustained release behavior compared with MFC. Also, it showed good stability for up to 90 days. Furthermore, a clinical appraisal of patients with melasma confirmed the superiority of the cream compared to the other cream in clinical study. **Conclusions**: The optimum OASPs present a promising approach for the treatment of melasma topically.

## 1. Introduction

Melasma is a widespread condition with complex pathogenesis and treatment. Navigating current and future therapy approaches requires an understanding of the pathophysiology and skin geography of melasma. Hypermelanosis of the skin that causes symmetrical brown-gray spots on the face is known as melasma [1].

UV radiation, hormones, oxidative stress, inflammation, vascular changes, and new signs of insulin resistance are all contributing factors to melasma, a multifactorial hyperpigmentation condition. By activating the PI3K/Akt and MAPK signaling pathways and raising tyrosinase activity, hyperinsulinemia may promote melanocyte proliferation and melanogenesis [1].

Many strategies have been adopted for treating melasma, including mechanical-based approaches such as chemical peels, physical therapies in the form of intense pulsed light sources or various types of lasers, as well as dermabrasion and microneedling. In addition, photoprotective measures such as avoiding exposure to direct sunlight and the regular application of sunscreens are constantly recommended. Camouflage cosmetics are used only to conceal the pigmentation spots, but not for the purpose of treatment. Moreover, oral remedies, as well as topical single or combination therapies and advanced topical nanodelivery systems, may aid in reducing hyperpigmentation. Advanced topical nanotechnology-based delivery systems can be employed to enhance the therapeutic effects of the topical hypopigmenting agents in prophylaxis and treatment [2].

Nanotechnology-based treatment strategies have immense potential to improve the therapeutic efficacy of anti-hyperpigmentation drugs for exclusive therapy of hyperpigmentation. The encapsulation of topical hypopigmenting drugs within nanocarrier-based delivery systems has been frequently explored recently for the effective management of melasma. These nanocarrier strategies have numerous advantages, which include enhanced drug permeation, drug targeting, improved therapeutic potential, stability against degradation, and rapid and prolonged action [3].

Ascorbic acid, also known as vitamin C, is an antioxidant that can bind to copper and successfully inhibit the tyrosinase enzyme. Therefore, it can suppress the oxidative polymerization of melanin intermediates. Consequently, melanin production in the melanogenesis process would be inhibited through ascorbic acid administration. As ascorbic acid acts as a depigmenting agent could be well-tolerated with minimal risk of irritation [4].

Several fatty ester derivatives of ascorbic acid were synthesized to transfer the peculiar antioxidant properties of ascorbic acid in lipophilic media and to improve its stability. All of them retained the antioxidant property of the ascorbyl moiety [5].

Another derivative from ascorbic acid is known as ascorbyl palmitate; with the addition of a lipid-soluble group, it might be expected that ascorbyl palmitate should provide a marked increase in ascorbic acid levels in the skin compared to topically applied ascorbic acid alone [6].

Aspasomes (ASPs) or ascorbyl acid-containing nanocarriers [7] are multilayered vesicles formed by amphiphile molecules having antioxidant activity, mainly ascorbyl palmitate, in combination with lipids (phospholipid and/or cholesterol) [8]. The ASPs have been utilized to enhance the skin deposition of various bioactives such as tizanidine [9], ferulic acid [10], and caffeic acid combined with retinoic acid [11].

A well-known oral anti-diabetic medication in the biguanide class is Metformin hydrochloride (MFC). It has been used as a primary treatment for type 2 diabetes mellitus, particularly when resources are scarce or when patients do not have cardiovascular or renal issues [12]. However, because of their additional cardiovascular and renal benefits, current international guidelines increasingly propose sodium glucose cotransporter-2 inhibitors or glucagon-like peptide-1 receptor agonists as the preferred first-line therapy [13]. Despite this change, MFC is still popular because of its pharmacological properties, which go beyond glycemic control [14]. According to earlier research, MFC has anti-inflammatory, anti-fibrotic, antioxidant, anti-apoptotic, and autophagy-inducing properties, all of which may be advantageous in dermatological applications, especially when used topically [15]. Moreover, previous research confirmed the ability of MFC to effectively manage melasma and hyperpigmentation [16]. Additionally, by lowering circulating insulin levels and enhancing insulin resistance, MFC may have therapeutic benefits for melasma by restricting melanocyte stimulation. Furthermore, MFC can repress MITF expression, downregulate mTOR signaling, and lower tyrosinase activity by activating AMP-activated protein kinase (AMPK), which reduces melanin formation.

To our knowledge, there have been no studies examining ASPs as a nanocarrier for the management of melasma; therefore, this study aimed to optimize ASPs to enhance MFC topical retention and to evaluate their safety and efficacy. In addition, the key point supporting the novelty of this work is not simply the use of ascorbic acid derivatives, but the selection of ASPs as a functional nanocarrier system. Unlike conventional liposomes, ASPs are vesicular systems in which ascorbyl palmitate (AP) itself acts as a structural component of the bilayer, not merely as an encapsulated active. This provides two main advantages: intrinsic antioxidant activity of the carrier, offering additional protection against oxidative stress involved in melasma pathogenesis. Using a D optimal mixture design, we investigated three formulation factors—Phosphatidyl choline (PC) amount (X_1_), AP amount (X_2_), and the presence or absence of ethanol (X_3_)—and measured their effects on entrapment efficiency (EE%; Y_1_), particle size (PS; Y_2_), polydispersity index (PDI; Y_3_), and zeta potential (ZP; Y_4_). We also assessed the effect of storage over time. Further, a differential calorimetry study was performed to assess the ability of ASPs to entrap MFC. Further, in vivo experiments in a clinical study assessed the therapeutic efficacy and safety of the formulations.

## 2. Materials and Methods

Metformin hydrochloride (MFC) was kindly provided by CID Pharmaceutical Co. (Giza, Egypt). Ascorbyl palmitate (AP) and phosphatidylcholine (PC) from egg yolk were purchased from Sigma-Aldrich Chemical Co. (St. Louis, MO, USA). Chloroform, ethanol, and methanol were obtained via El-Nasr Chemicals Co. (Cairo, Egypt).

### 2.1. Methods

#### 2.1.1. Experimental Design

For an adequate estimation of the factors’ effects, MFC-loaded ASPs were optimized via a three-factor D-optimal design, using Design-Expert^®^ 13 software (Stat-Ease, Inc., Minneapolis, MN, USA). The design contained two numeric (continuous) factors, namely PC and AP amounts in mg. The third factor was a categorical (nominal) factor and studied the effect of the presence/absence of ethanol in the final formulation. The search within the design was conducted through point exchange, including three replicate points and three lack-of-fit points, resulting in fifteen experimental runs. Percentage entrapment efficiency (%EE), particle size (PS), polydispersity index (PDI), and absolute value of zeta potential (ZP) were recorded as the observed responses. For optimization, it was desirable to maximize both %EE and ZP and to minimize PS and PDI. Table 1 compiles the factors, their upper and lower levels, the observed responses, and their desirability constraints [17]. The experimental design is presented in Table 1; its factors and levels were determined based on preliminary screening studies.

#### 2.1.2. Metformin Hydrochloride Aspasome (ASP) Fabrication

ASPs were fabricated using the thin-film hydration method using various amounts of both PC and AP. Firstly, PC and AP were dissolved in a 10 mL organic solvent mixture of chloroform and methanol (7:3 *v*/*v*). The mixture was allowed to rotate at 60 °C (which is above the lipid phase transition temperature; this temperature is below the reported degradation threshold of the active pharmaceutical ingredient) and 150 rpm (Rotavapor, Heidolph VV 2000, Burladingen, Germany) until the formation of a thin and dry film. The film was then hydrated using MFC (10 mg) solution in either 10 mL of double-distilled water or water containing ethanol at 1% *v*/*v* concentration from the total volume, followed by rotation for 45 min at the same temperature and speed. After that, dispersions were kept at 4–8 °C till further investigation [18].

#### 2.1.3. Estimation of the Percentage Entrapment Efficiency

The indirect method was adopted for the estimation of the %EE of MFC within ASPs. In brief, 1 mL of ASPs dispersion was centrifuged at 22,000 rcf and 4 °C for 60 min in an ultracentrifuge (3 K30, Sigma, Steinheim, Germany). Afterwards, the supernatant, containing unentrapped MFC, was suitably diluted and spectrophotometrically analyzed at λ_max_ of 233 nm [19]. The %EE was calculated from the following equation:

% EE = ((Total drug content − unentrapped amount)/(Total drug content)) × 100(1)

#### 2.1.4. Particle Size (PS), Polydispersity Index (PDI), and Zeta Potential (ZP) Measurements

Briefly, 100× dilutions of ASPs in double-distilled water were utilized for measuring PS and PDI of ASPs, using a zetasizer (Model ZEN3600, Malvern Instruments Ltd., Worcestershire, UK). Employing a special cuvette with the same equipment, ZP was measured, and the absolute values were then used in the optimization process [20]. To ensure the reproducibility of results, measurements were conducted in triplicate.

#### 2.1.5. Application of Desirability Criterion for Optimization of ASPs

Employing the numerical optimization function in Design-Expert^®^ 13 software, the design constraints listed in Table 1 were fed into it, and the solution with the highest composite desirability was suggested as the optimum ASP formulation (OASP), which was then fabricated using the same method. Subsequent comparison of the observed and predicted responses of OASPs via the calculation of bias percentage was conducted through the following equation:

Bias percentage = (|Predicted value − Observed value|/(Observed value)) × 100(2)

Afterwards, OASPs were subjected to extensive in vitro and in vivo assessments.

### 2.2. In Vitro Testing of OASPs

#### 2.2.1. Transmission Electron Microscopy (TEM)

To elucidate the morphology of OASP vesicles, and to confirm the validity of zetasizer-derived PS, a transmission electron microscope (JEOL, Tokyo, Japan) was employed at 80 kV after the dilution of one drop of OASPs, and its subsequent drying on a carbon-coated copper grid and staining with 2% *w*/*v* phosphotungstic acid [21].

#### 2.2.2. Lyophilization and Differential Scanning Calorimetry (DSC)

The OASP was frozen and then lyophilized using a lyophilizer (Novalyphe-NL 500, Savant Instruments, New York, NY, USA) for an entire day at −45 °C and a 7 × 10^−2^ mbar pressure. MFC and lyophilized OASPs thermograms were recorded, using a previously calibrated differential scanning calorimeter (Shimadzu DSC 50; Kyoto, Japan) after heating 3–4 mg samples of each in hermetically sealed flat-bottomed aluminum pans over a temperature range of 30 to 250 °C, at a constant heating rate of 10 °C/min, under an inert nitrogen flow of 30 mL/min [22].

#### 2.2.3. In Vitro Release Study of MFC from OASPs

USP dissolution apparatus II (Pharma Test, Hainburg, Germany) was utilized to assess in vitro MFC release from OASPs and an aqueous solution of the same concentration for 6 h at 37 °C. An amount of 1 mL of OASPs or MFC aqueous solution (corresponding to 1 mg of MFC) was put into plastic tubes with a 3.14 cm^2^ permeation area, with one end firmly sealed with a cellulose membrane and the other end linked to the shaft of the dissolution equipment rather than the baskets. The receptor medium was 50 mL of phosphate buffer (pH 5.5). Aliquots were withdrawn at 1, 2, 3, 4, 5, and 6 h. The samples were examined by applying a UV spectrophotometer set to λ_max_ 233 nm. The measurements were conducted in triplicate. Release profiles were obtained by plotting percentage released vs. time, and plot points were represented as the mean percentage released ± standard deviation [23].

#### 2.2.4. Effect of Short-Term Storage

The effect of short-term storage on OASPs was studied by refrigerating OASPs at 2–8 °C for 90 days. Afterwards, OASPs were visually inspected for changes in their morphology (color, homogeneity, ease of redispersion, and appearance of visible aggregates). Also, the measured responses (%EE, PS, PDI, and ZP) of fresh and stored OASPs were compared via Student’s *t*-test, setting α at 0.05.

### 2.3. In Vivo Clinical Assessment of OASPs

#### 2.3.1. Patient Recruitment

A double-blind split-face study was conducted in the dermatology outpatient clinic of Minia University Hospital. The clinical assessment was approved by the Ethical Committee for Postgraduate Studies and Research, Faculty of Medicine, Minia University (Approval No. 926/10/2023), and was conducted in accordance with the Declaration of Helsinki. The study included ten female melasma patients who provided informed consent before participating in the study. The clinical application and assessments were performed under the supervision of a qualified dermatology consultant in a hospital clinical setting. During clinical assessment visits, patients were evaluated for treatment response, compliance, and tolerability. All participants were also instructed to report any local or systemic adverse drug reactions during the study period. The developed formulation was intended for topical use, and all its components, including phosphatidylcholine, ascorbyl palmitate, ethanol, and metformin, have well-established safety profiles in pharmaceutical and topical applications. Furthermore, prior to clinical use, the formulation underwent extensive physicochemical characterization, stability testing, and in vitro evaluation to ensure its quality and suitability for clinical application. Patients had a mean age of 31.5 years (ranging from 18 to 42), and a mean disease duration of 24.9 months (Table 2). The skin phototypes of patients included in the study ranged from type III to type IV according to Fitzpatrick’s classification, characterized by skin tones ranging from white to lightly tanned, veering towards a tanned skin color [24]. The patients were treated on the right side of the face with optimum MFC-loaded OASP, while the left side was treated with Kligman’s formula (prepared in a local drug store as an aqueous (*o*/*w*) cream containing 2% hydroquinone, 1% mometasone furoate, and 0.025% tretinoin) [25] for two months, with both treatments applied topically in the form of a thin film once at night.

#### 2.3.2. Evaluation of Modified Melasma Area Severity Index (mMASI) and Physician Global Assessment (PGA)

To assess the efficacy of treatments in reducing melasma severity, the modified Melasma Area Severity Index (mMASI) was evaluated at baseline and after two months of treatment, followed by the calculation of the reduction percentage with its grading by physician global assessment (PGA), and patient global assessment (PtGA) where the improvement was reported as mild (0–25%), moderate (26–50%), good (51–75%), or excellent (76–100%), for each patient on each side of the face. Additionally, the physician global assessment (PGA) of melasma was evaluated 2 months after the treatment initiation of both regimens to assess the overall clinical response as judged by the physician. The PGA offers a complementary qualitative method to the mMASI score, as it is a standardized, clinician-rated global evaluation tool used to assess visible improvements in pigmentation and lesion size. The mMASI scores at baseline and after treatment for each treatment, as well as the comparison between the two treatments, were conducted using paired and independent-samples *t*-tests, respectively. The PGA scores after two months were compared between the two treatments using the Chi-square test. IBM’s SPSS^®^ software V24 was used for statistical analysis.

#### 2.3.3. Immunohistochemical Evaluation and Histopathologic Assessment

Histopathologic examination of skin biopsies obtained as 1 mm punches from the melasma areas on each side of the face was done at the baseline and after 2 months of treatment. These biopsies were sectioned and stained by hematoxylin and eosin (H&E) and immunohistochemically using a monoclonal mouse anti-melanoma antigen recognized by T cells-1 (MART-1) (Melan A, Clone A 103, monoclonal antibody, diluted 1:50; Dako, Santa Clara, CA, USA). A light microscope [Accu-Scope # 3025 five-headed (A3025-5); Olympus, Tokyo, Japan] with a built-in camera (digital camera E-330SLR; Olympus) was used to examine and photograph the sections. Interpretation of the immunohistochemical marker was performed by two blinded independent dermatopathologists through counting the number of positive (active) melanocytes in the epidermis and dermis (melanophages) in 10 high-power fields with measurement of the mean value for each biopsy.

## 3. Results and Discussion

### 3.1. Metformin Hydrochloride Aspasome (ASP) Fabrication

ASPs were successfully prepared using the thin-film hydration method, with the PC and AP contents responsible for the faint yellow color. The adopted D-optimal design resulted in fifteen formulations (ASP1–ASP15), whose compositions and measured responses are recorded in Table 3. The same table also includes the PDI measurements for different formulations, which ranged from 0.420 ± 0.003 to 0.675 ± 0.002, indicating a minor degree of heterogeneity in PS, yet still within the valid range for its estimation through dynamic light scattering [26].

#### 3.1.1. Experimental Design Interpretations

Table 4 reveals that the Design-Expert^®^ software suggested different models for the analysis of various responses. The closeness between adjusted and predicted R2 values (differences smaller than 0.2) for all the responses studied concluded the models’ ability to navigate design space and predict the corresponding responses.

#### 3.1.2. %EE Model Analysis

**Figure 1:**
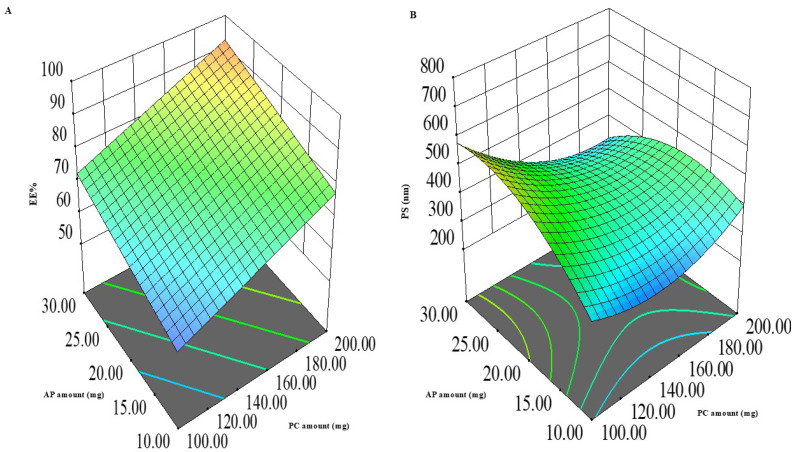
Three-dimensional plot for the impact of AP amount (X_1_) and PC amount (X_2_) on EE% (**A**) and PS (**B**). AP: Ascorbyl palmitate; PC: phosphatidyl choline; EE%: entrapment efficiency percent; PS: particle size.

From Table 3, it could be seen that the encapsulation of MFC in ASPs was successful, with %EE values ranging from 54.9 ± 1.1 to 96.3 ± 2.3%. Figure 1 and the ANOVA output in Table 4 demonstrate that both X_1_: PC amount and X_2_: AP amount had a significantly positive effect on %EE (*p* < 0.0001 for both), while X_3_: the absence/presence of ethanol had a significant negative effect, where the presence of ethanol decreased %EE (*p* = 0.0002).

The positive relation between the PC amount and %EE can be explained because PC is the main vesicle former in the system. Therefore, increasing its amounts leads to the formation of a larger number of vesicles, providing more intravesicular spaces for the entrapment of MFC, retarding its diffusion outside the vesicles [27,28]. The finding is consistent with many studies in the literature that confirm the positive impact of PC on the %EE of different drugs. Further, previous studies by Fahmy et al., 2025 [29], and Jahanfar et al. [30] positively correlated the PC amounts and the %EE upon the preparation of curcumin PEGylated terpesomes, propranolol hydrochloride PLGA hybrid nanoparticles, and green tea extract liposomes, respectively [24].

The positive relation between AP and %EE can be attributed to the fact that AP itself is reported as an adjunctive vesicle former; it can incorporate itself into the vesicular bilayers, leading to the formation of a larger number of vesicles that can entrap larger amounts of MFC. Additionally, AP is reported to be suitable for the entrapment of hydrophilic drugs [7]. The results are in line with those obtained by Aboul-Einien et al. [8], upon the preparation of ASPs of the hydrophilic magnesium AP.

The presence of ethanol in the formulation is favorable due to its modification of vesicular membrane rigidity, allowing for drug entrapment [27]. However, there was a negative impact of the presence of ethanol in ASPs on %EE, which could be attributed to the fluidizing effect of ethanol on the vesicular membranes and the possible pore formation, leading to leakage of the entrapped drug [31]. This agrees with the findings of Wang and Huang [32] and Raghav et al. [31], where the encapsulation of proteins in liposomes and kaempferol in ethosomes was decreased by increasing ethanol concentration in the final formulation.

#### 3.1.3. PS Model Analysis

From Table 3, it can be seen that the PS of ASPs ranged from 231.5 ± 0.5 to 705.0 ± 2.0 nm. Figure 1 and the ANOVA output in Table 4 demonstrate that both X_1_: PC amount and X_2_: AP amount were significantly inversely related to PS, with *p* values of 0.0019 and 0.015, respectively. Also, multiple interactions were significant. These included the AB interaction between PC and AP amounts, the AC interaction between PC amount and absence/presence of ethanol, and the BC interaction between AP amount and absence/ presence of ethanol (*p* = 0.0004, 0.0108, and 0.0006, respectively). In all these interactions, the mutual navigation from the low to high levels of factors synergistically decreased PS. The inverse relation between PS and the PC and AP amounts can be related to the surface activity of PC and AP (HLB = 7 and 8.4, respectively). At higher amounts of both, there is a tendency to reduce the interfacial tension at the lipid/water interface, enhancing surface curvature and maintaining steric stabilization, thus forming smaller vesicles that do not tend to coagulate [33]. Similar findings were observed by Ammar et al. [27], where increasing PC concentration led to smaller vardenafil hydrochloride invasomes. Although ethanol presence by itself did not have a significant effect on PS, it helped both PC and AP reduce PS through similar mechanisms, as evident by the significant AC and BC interactions [34,35]. Similar findings were obtained by Galatage et al. [36] during the preparation of Acacia Senegal and ropivacaine ethosomes, respectively.

#### 3.1.4. PDI Model Analysis

**Figure 2:**
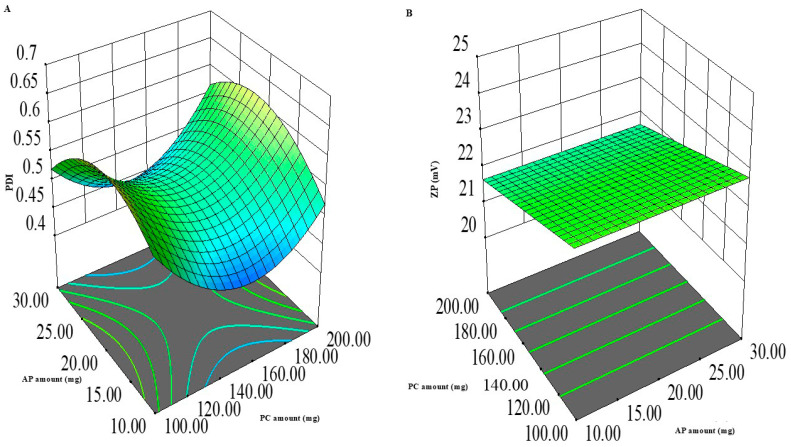
Three-dimensional plot for the impact of AP amount (X_1_) and PC amount (X_2_) on PDI (**A**) and ZP (**B**). AP: Ascorbyl palmitate; PC: phosphatidyl choline; PDI: polydispersity index; ZP: zeta potential.

From Table 3, it could be seen that the PDI values of ASPs ranged from 0.420 ± 0.003 to 0.675 ± 0.002, indicating a degree of heterogeneity in PS, yet still inside the valid range for its estimation through dynamic light scattering [37]. Figure 2 and the ANOVA output in Table 4 demonstrate that only X_3_: the absence/presence of ethanol was significant, where the presence of ethanol significantly reduced PDI values (*p* < 0.0001). However, multiple significant interactions were observed. The AB interaction between PC and AP synergistically increased the PDI values in the absence of ethanol, while the opposite was observed in its presence; a net decrease in PDI was observed (*p* = 0.0018). Finally, the AC and BC interactions were significant, where ethanol counteracted the increase in PDI caused by increasing both PC and AP amounts (*p* = 0.0065 and <0.0001, respectively).

The desirable effect of the presence of ethanol on PDI is attributed to its surface activity and its ability to favor the formation of more uniformly sized vesicles [38]. Similar observations were obtained by Wang et al. [32], where increasing ethanol concentration led to the formation of more uniform kaempferol ethosomes.

#### 3.1.5. ZP Model Analysis

As all ZP values were in the negative range, these were expressed as absolute values for clarity. From Table 3, it could be seen that the ZP values of ASPs ranged from −20.0 ± 1.0 to −24.6 ± 0.4 mV (absolute value). Although it is preferable that colloidal dispersions have a ZP value greater than ±30 mV for better physical stability and reduced aggregate formation [39], the values presented in this study are not very far from the desired range. Other studies in the literature reported the formation of stable colloidal dispersions with ZP values lower than the mentioned range, depending on other factors other than the absolute surface charge. Albash et al. [40] obtained a stable optimized cationic ceramide/phospholipid composite of levocetirizine hydrochloride with a ZP value of 20.2 ± 1.1 mV through the elevated medium viscosity and enhanced steric stabilization offered by ceramide III. Additionally, Fahmy et al. [41] obtained a stable ternary micellar system of voriconazole that has a ZP value of −9.0 mV through the stabilization of micelles by the projected polyethylene glycol and polyethylene oxide chains of the employed Pluronic. Here, in our study, Figure 2 and the ANOVA output in Table 4 reveal that both X1: PC amount and X3: absence/presence of ethanol had a significant effect on the ZP of ASPs, where increasing the amount of PC and the presence of ethanol significantly increased the ZP values of ASPs (*p* = 0.0026 and 0.0016, respectively). Additionally, the AC interaction between PC amount and absence/presence of ethanol and the BC interaction between AP amount and absence/presence of ethanol were significant (*p* < 0.0001 and *p* = 0.0031, respectively). The presence of ethanol promoted the negative ZP upon increasing AP amounts, while a reduction was noted upon increasing PC amounts. However, all the values were close to each other, as mentioned before (Table 3).

Regarding the augmenting effect of PC amount and the presence of ethanol on ZP, the use of higher PC amounts in an aqueous medium of weak ionic strength would promote the projection of the negatively charged phosphatidyl group of the polar head outwards, while the positively charged choline group would be directed inwards, promoting a net negative surface charge [42]. Similar findings were observed by [43] upon studying the effect of pH and ionic strength on phospholipid nanomechanics.

This effect is augmented by the inclusion of ethanol that carries a partially negative charge that would stabilize the system by enhancing electrostatic repulsion and preventing vesicular aggregation [44]. Albash et al. [45] obtained similar results during the preparation of olmesartan medoxomil transethosomes.

### 3.2. Application of Desirability Criterion for Optimization of ASPs

By maximizing %EE and ZP (absolute values), and minimizing PS and PDI, the Design-Expert^®^ software suggested an optimized ASP formulation (OASP), which had the highest composite desirability (0.890) and contained 193.12 and 30 mg of PC and AP, respectively, in the presence of ethanol. The observed and predicted responses of OASP are compared in Table 4. From the small bias percentages calculated, the ability of the design to accurately predict different responses was concluded. OASP was then extensively tested both in vitro and in vivo.

### 3.3. In Vitro Testing of OASP (Physicochemical Properties)

#### 3.3.1. Transmission Electron Microscopy (TEM)

**Figure 3:**
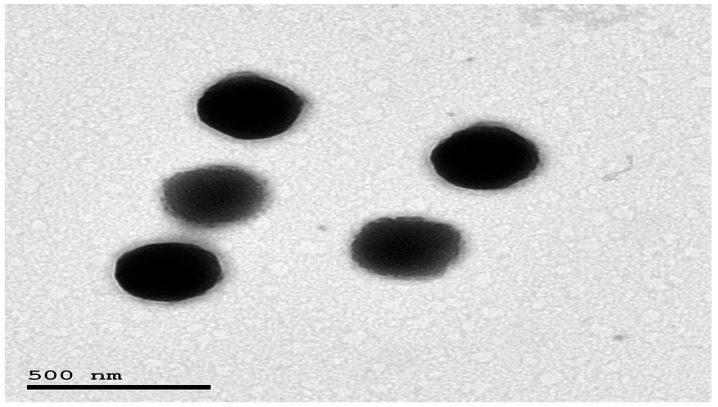
Morphological structure of the OASP.

Figure 3 demonstrates the spherical morphology of OASP. Also, the PS was near that obtained via the dynamic light scattering technique (zetasizer), validating its PS determinations [21].

#### 3.3.2. Differential Scanning Calorimetry (DSC)

**Figure 4:**
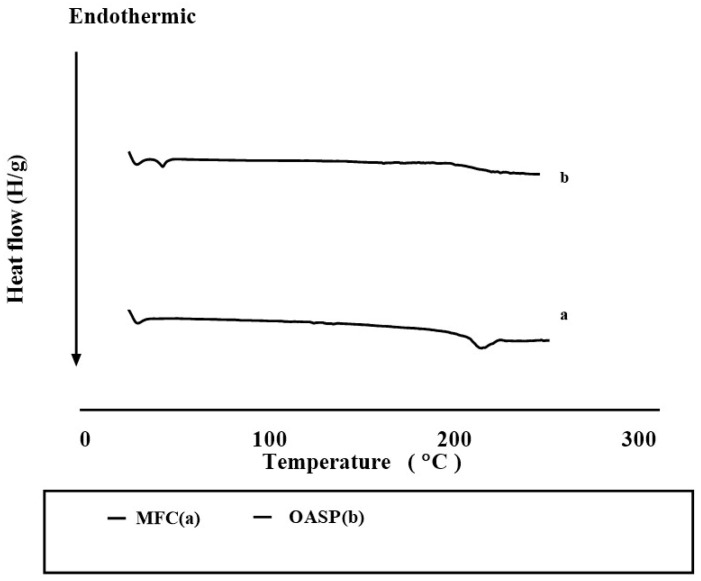
DSC thermograms of MFC (**a**) and the optimal OASP (**b**).

Figure 4 shows the sharp endothermic peak of pure MFC at 229.8 °C, corresponding to its melting point. The disappearance of such a peak in the thermogram of OASP could be attributed to the loss of crystallinity or the dilution effect in the formulation [46].

### 3.4. In Vitro Release Study of MFC from OASP

**Figure 5:**
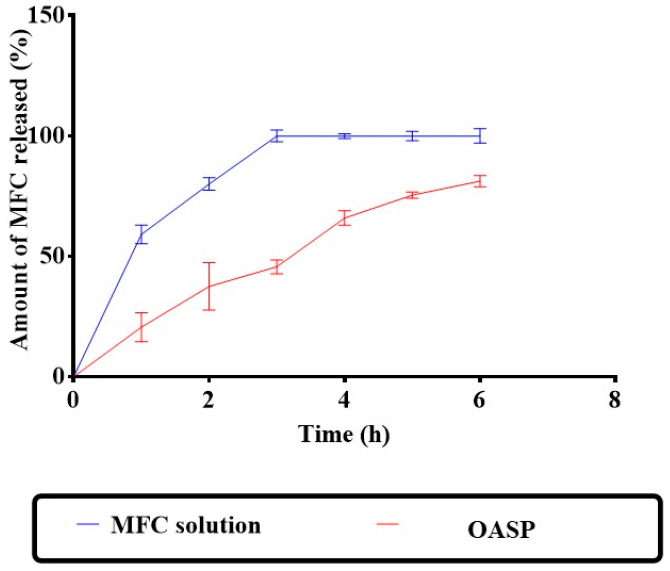
In vitro release data of the drug solution and the optimal aspasomes.

Being a water-soluble drug, the MFC solution released all its MFC content in just 3 h, with about 80% released in the first 2 h (Figure 5). In contrast, OASP released only about 38% of its MFC content in the first 2 h and maintained its sustained release behavior for the rest of the study, releasing about 80% at 6 h. These results confirm the effective encapsulation of MFC inside OASP vesicles, with a greater time needed for MFC to diffuse across the hydrophobic barriers of OASP vesicles. Another reason for the observed results is the higher medium viscosity of OASP, compared to the MFC aqueous solution [47]. Similar results were obtained by Ahmed et al. [48] and Albash et al. [49] upon the preparation of terconazole proniosomes and propranolol hydrochloride PLGA–lipid hybrid nanoparticles, respectively.

#### Effect of Short-Term Storage

After refrigerating OASP at 2–8 °C for 90 days, OASP preserved its morphology. It retained its original color, homogeneity, and was easily redispersible, with no visible aggregates. The results for the stored OASP were EE% of 86.10 ± 0.130, PS of 274.82 ± 0.02 nm, PDI of 0.433 ± 0.003, and ZP of −20.77 ± 0.02 mV, respectively. Also, Student’s t-test for the measured responses (%EE, PS, PDI, and ZP) of fresh and stored OASP at α of 0.05 yielded non-significant results, with all *p* values > 0.05. All the previous observations concluded with the stability of OASP under the specified storage conditions.

### 3.5. In Vivo Clinical Assessment of OASP

#### Modified Melasma Area Severity Index (mMASI), Physician (PGA), and Patient Global Assessment (PtGA) Evaluations

Available evidence from multiple human trials indeed shows the clinical benefit of topical MFC in the treatment of melasma and decreasing mMASI score, being comparable to Kligman’s formula, which is considered the standard treatment of melasma [15]. Interestingly, in the present study, the percentage of reduction in mMASI on the right side (OASP) was significantly higher than on the left side (Kligman’s formula) (61.09% vs. 40.01%, respectively, *p* < 0.001) after two months (Table 5).

**Figure 6:**
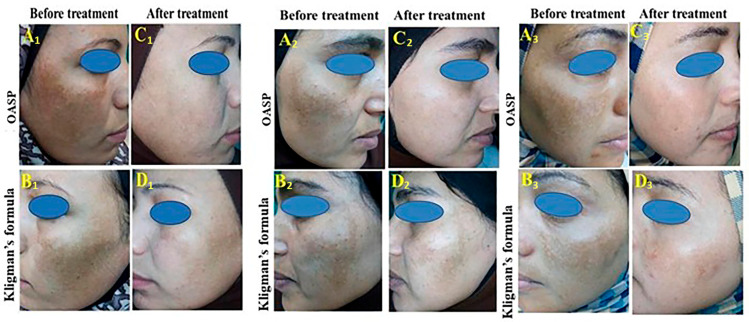
Representative clinical photographs of three female patients with epidermal melasma presenting as brown patches over the malar region on both sides of the face before and after treatment. For each patient, the right side of the face was treated with the optimal MFC-loaded aspasomal formulation (OASP), while the left side was treated with Kligman’s formula. (**A1**–**A3**) Right side before treatment with OASP. (**B1**–**B3**) Right side after 2 months of treatment with OASP. (**C1**–**C3**) Left side before treatment with Kligman’s formula. (**D1**–**D3**) Left side after 2 months of treatment with Kligman’s formula. The OASP-treated side showed greater clinical improvement compared with the Kligman-treated side.

Two months after study initiation, the physician global assessment (PGA) of the OASP-treated side was significantly improved compared to the Kligman-treated side (Table 6). Specifically, 60% of patients receiving OASP (right side of the face) showed excellent improvement, and 40% showed good improvement (Table 6, Figure 6). In contrast, in the Kligman’s formula-treated side, no cases showed excellent improvement, 70% showed good improvement, and 30% showed moderate improvement (Table 6, Figure 6). This finding confirms the superiority of the MFC-based ASP formula to Kligman’s treatment and is consistent with the trends observed in mMASI scores. Furthermore, no serious adverse effects or treatment discontinuations were reported during the study period, confirming the tolerability of the developed formulation.

### 3.6. Immunohistochemical Evaluation and Histopathologic Assessment

**Figure 7:**
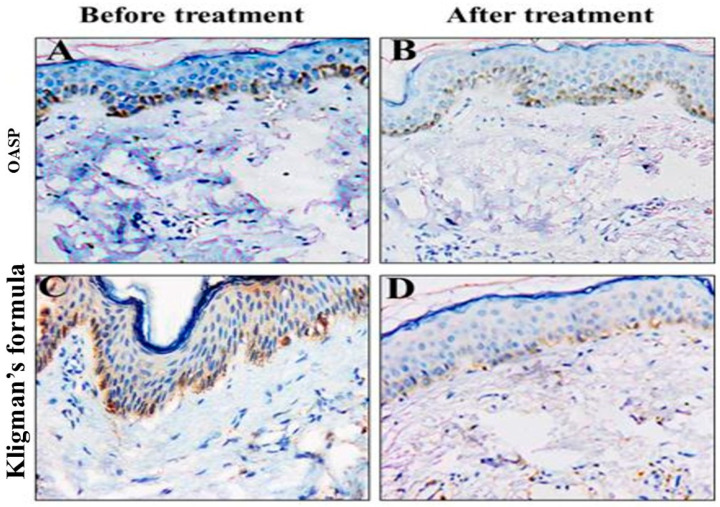
Two groups comprising skin biopsies from epidermal melasma lesions before and after treatment: (**A**) Right side of the face before optimal MFC-loaded aspasomal formulation (OASP) treatment. (**B**) Right side of the face after OASP treatment. Biopsies from the right side of the face after 2 months of treatment with MANs (**B**) demonstrated a notable decrease in the number of epidermal melanoma antigen recognized by T-cells 1 (MART-1)-positive cells. (**C**) Left side of the face before Kligman’s formula treatment. (**D**) Left side of the face after Kligman’s formula treatment. Biopsies from the left side of the face after 2 months of treatment with Kligman’s formula (**D**) also demonstrated a decrease in the number of epidermal MART-1-positive cells. However, the reduction appears greater in the MANs-treated group compared to the Kligman’s formula group (MART-1 stain, X200).

**Figure 8:**
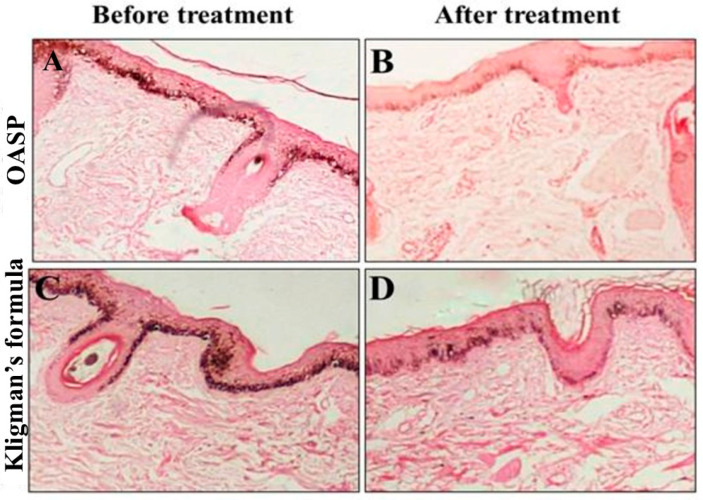
Two groups comprising skin biopsies from epidermal melasma lesions before and after treatment: (**A**) Right side of the face before optimal MFC-loaded aspasomal formulation (OASP) treatment. (**B**) Right side of the face after OASP treatment. (**C**) Left side of the face before Kligman’s formula treatment. (**D**) Left side of the face after Kligman’s formula treatment. Biopsies from the right side of the face after 2 months of topical application of MANs treatment (**B**) demonstrated a remarkable decrease in epidermal Melanin Particle Surface Area (MPSA). While biopsies from the left side of the face after 2 months of topical application of Kligman’s formula treatment (**D**) demonstrated a less notable decrease in epidermal MPSA. The reduction in MPSA appears greater in the MANs group compared to the Kligman’s formula group (MF stain, X200).

Quantitative immunohistochemical analysis of skin biopsies revealed that both treatment regimens induced a statistically significant decrease in melanoma antigen recognized by T-cells 1 (MART-1)-positive cells in the dermis and epidermis layers after two months of daily application on both the right and left sides of the face (Table 7). Notably, the right side of the face where the OASP was applied showed a more pronounced (*p* < 0.05) decrease in the number of MART-1-positive cells within the dermis and epidermal layers compared to the left side of the face where Kligman’s formula was applied (Table 7). Also, the total percent reduction in MART-1-positive cells was more substantial on the OASP side, with a decrease of 63.64%, compared to 36.61% on the Kligman’s formula side (*p* = 0.0002). This finding was confirmed by histological observations from skin biopsies, as illustrated in Figure 7. This figure depicts the changes in epidermal melasma lesions before and after treatment. Specifically, biopsies from the right side of the face treated with OASP showed a more pronounced reduction in the number of MART-1-positive cells than the left side, which was treated with Kligman’s formula, consistent with the quantitative data. Further, the OASP formula has shown greater ability in decreasing Melanin Particle Surface Area (MPSA) after two months of treatment application, in comparison to Kligman’s formula, as shown in Figure 8.

Overall, histopathological examination of skin biopsies confirmed that treatment with topical OASP and Kligman’s formula significantly reduced MART-1-positive melanocyte counts in both epidermal and dermal melasma lesions after two months of daily application, with the OASP formula achieving a markedly greater reduction in the total percentage of MART-1-positive cells in the dermis and epidermis layers and also in MPSA, compared to Kligman’s formula. Both are critical immunohistochemical markers for evaluating melasma treatment efficacy, where MART-1 reflects melanocyte activity, while MPSA measures melanin density, both pivotal elements in melasma pathogenesis [4].

Kligman’s formula, composed of hydroquinone, tretinoin, and a corticosteroid, is considered the gold standard in the treatment of melasma by inhibiting melanogenesis and promoting epidermal turnover [50]. However, our study demonstrated that Kligman’s formula significantly decreases the number of MART-1-positive cells, which indicates direct suppression of melanocyte activity. This finding was supported by a previous study reporting that hydroquinone 4% cream, a major component of Kligman’s formula, significantly reduces melanin content in the basal epidermis by inhibiting tyrosinase. This, in turn, leads to a reduction in melanocyte activity and clinical lightning of pigmentation [16]. Additionally, the present study provides novel insights into the mechanism of MFC in the treatment of melasma. While previous studies have indicated that inhibiting melanogenesis by downregulating microphthalmia-associated transcription factor-MITF expression and suppressing melanocyte proliferation is the primary mechanism of MFC in the treatment of melasma [51], the histological (Figure 8) examination of our study suggested an additional pathway, which was the modulation of melanocyte activity and density. This is evidenced by a reduction in MART-1-positive cells and structural changes in the basal layer of the epidermis following MFC topical application. These results indicate a dual-action therapeutic effect of MFC, both suppressing melanin production and altering melanocyte activity and distribution within the skin.

Regarding a direct comparison between the topical OASP and Kligman’s formula, it was previously reported that they possess similar efficacy in treating melasma, with MFC being the safer option [52]. The present study demonstrated that OASP was superior to Kligman’s formula, where OASP reduced melanocyte numbers and activity without notable epidermal damage or inflammation, as observed histologically. This could be attributed to the novel formula used in the present study, namely, an aspasomal nanoparticle formulation of MFC. The vesicles showed high EE% (87.50 ± 0.33%), ensuring effective drug loading for topical delivery. The PS (264.47 ± 0.02 nm) is suitable for retention in the upper epidermal layers, enhancing localized effect while limiting systemic absorption. The moderately negative ZP (−21.67 ± 0.12 mV) provides sufficient electrostatic repulsion to maintain stability without compromising skin compatibility. Moreover, the fabricated ASPs’ small PS and high EE% overcame the common limitations of standard treatments, such as poor skin penetration and rapid drug degradation. Collectively, these properties support the clinical relevance of the vesicles for safe and effective topical therapy.

This formula could strengthen MFC ability in the treatment of melasma and affect melanocytes by offering several advantages: the lipid-based nanoform enhances skin penetration, allowing MFC to affect pigment in deeper layers, and it provides sustained drug release, maintaining therapeutic levels for longer periods, hence reducing the frequency of application [53]. While ASP encapsulation of MFC enhances its stability and bioavailability by preventing MFC from degradation on the skin surface [54]. This targeted-delivery system minimizes systemic exposure, thereby lowering the risk of side effects.

Despite the promising clinical and histopathological results of the present trial, the study has some limitations. The relatively small number of participants may limit the generalizability of the findings. However, the split-face design strengthened internal validity by reducing inter-individual variability, allowing each patient to serve as their own control. In addition, 1 mm punch biopsies were intentionally selected to reduce invasiveness, patient discomfort, and the risk of cosmetic scarring, particularly given the facial location of the lesions. Although small, these biopsies were sufficient for reliable immunohistochemical and histopathological evaluation. Another limitation of the present study is the absence of a metformin solution-treated control group, which may limit the ability to directly assess the specific contribution of the aspasomal carrier. The present study aimed to compare the metformin-loaded aspasomal formulation with the standard treatment, Kligman’s formula, to evaluate its clinical efficacy and therapeutic relevance. Previous studies have demonstrated improved efficacy of metformin nanocarrier systems compared with metformin solution. Therefore, future studies including a metformin solution control group as a comparator are recommended to provide additional mechanistic insights. Nevertheless, larger-scale randomized clinical studies with increased sample size and extended follow-up are recommended to further confirm the clinical efficacy and safety of the metformin-loaded aspasomal formulation in the treatment of melasma.

## 4. Conclusions

This study successfully studied metformin hydrochloride and enhanced its therapeutic performance through formulation into aspasomes. The optimum formulation showed high EE%, nanoscale size, uniform distribution, favorable surface charge, and excellent physical stability. The differential scanning calorimetry study proved the successful encapsulation of metformin hydrochloride inside aspasomes. Further, the optimum formulation showed a sustained effect of the aspasomes compared to the metformin hydrochloride solution. The superior efficacy in clinical appraisal using the optimal MFC-loaded aspasome (OASP) formulation likely stems from its optimized delivery, synergistic mechanisms, and ROS-scavenging properties, offering a novel, patient-friendly alternative to TCC. This explanation ties innovative formulation design to the observed clinical superiority, emphasizing how nanotechnology and combinatorial chemistry address the limitations of earlier approaches.

## Figures and Tables

**Table 1:** D-optimal mixture design for optimizing MFC-loaded ASPs.

Factors (Independent Variables)	Levels	
	Low (−1)	High (+1)
X_1_: PC amount (mg)	100	200
X_2_: AP amount (mg)	10	30
X_3_: Absence/presence of ethanol	Absent	Present
Responses (dependent variables)	Constraints	
Y_1_: %EE	Maximize	
Y_2_: PS (nm)	Minimize	
Y_3_: PDI	Minimize	
Y_4_: ZP (mV)	In range	

Abbreviations: ASPs: Aspasomes; MFC: metformin hydrochloride; PC: phospholipid; AP: ascorbyl palmitate; %EE: entrapment efficiency; PS: particle size; PDI: polydispersity index; and ZP: zeta potential.

**Table 2:** The basic characteristics of the included patients.

Characteristics	Value
Age (years)	
Mean ± SD	31.5 ± 8.41
(Range)	(18–42)
Sex, n (%)	
Female	10 (100%)
Skin type, n (%)	
III	5 (50%)
IV	5 (50%)
Family history, n (%)	
+ve	5 (50%)
−ve	5 (50%)
Histological type, n (%)	
Mixed	6 (60%)
Epidermal	4 (40%)
Clinical type, n (%)	
Centrofacial	1 (10%)
Malar	6 (60%)
Malar, Mandibular	3 (30%)
Duration of disease (months)	
Mean ± SD	24.9 ± 16
(Range)	(9–60)

Data are presented as mean ± standard deviation (SD), (interquartile range [25th–75th percentile] (IQR)), frequency, and percent.

**Table 3:** Results of D-optimal design of MFC-loaded ASPs formulations.

	PC Amount (mg)	AP Amount (mg)	Ethanol	%EE	PS (nm)	PDI	ZP (mV)
ASP1	100	10	Absent	61.19 ± 1.30	360.00 ± 1.00	0.580 ± 0.001	−21.5 ± 0.51
ASP2	100	10	Present	54.92 ± 1.12	315.61 ± 1.28	0.557 ± 0.018	−24.56 ± 0.43
ASP3	100	20	Present	60.33 ± 1.10	509.19 ± 2.34	0.550 ± 0.010	−24.12 ± 0.41
ASP4	100	30	Absent	75.00 ± 2.00	705.00 ± 2.00	0.658 ± 0.002	−20.00 ± 1.00
ASP5	100	30	Absent	75.00 ± 2.00	705.00 ± 2.00	0.675 ± 0.002	−20.00 ± 1.00
ASP6	150	10	Absent	67.31 ± 1.78	231.51 ± 0.53	0.420 ± 0.003	−22.90 ± 0.10
ASP7	150	10	Present	65.38 ± 1.78	374.19 ± 1.96	0.479 ± 0.002	−21.91 ± 0.09
ASP8	150	20	Absent	85.18 ± 2.32	427.23 ± 2.12	0.580 ± 0.001	−21.63 ± 0.12
ASP9	175	20	Present	76.12 ± 2.32	398.00 ± 2.00	0.470 ± 0.010	−22.00 ± 2.00
ASP10	200	10	Absent	81.71 ± 2.50	315.34 ± 3.65	0.49 ± 0.011	−23.11 ± 0.91
ASP11	200	10	Present	72.77 ± 3.64	483.18 ± 0.12	0.550 ± 0.005	−21.00 ± 1.00
ASP12	200	30	Absent	96.29 ± 2.31	355.23 ± 2.12	0.670 ± 0.003	−21.41 ± 0.12
ASP13	200	30	Present	88.00 ± 1.00	270.92 ± 0.01	0.453 ± 0.002	−21.33 ± 0.18
ASP14	200	30	Present	88.00 ± 1.00	270.91 ± 0.01	0.453 ± 0.017	−21.33 ± 0.15
ASP15	200	30	Absent	96.29 ± 2.31	355.23 ± 2.12	0.680 ± 0.020	−21.71 ± 0.13
Observed values for OASP				87.50 ± 0.3%	264.47 ± 0.02	0.423 ± 0.001	−21.67 ± 0.12
Predicted values for OASP				87.49	264.30	0.420	−21.60
Bias (%)				0.01	0.06	0.70	0.18

Abbreviation: MFC: Metformin hydrochloride; ASPs: aspasomes, PC: phospholipid; AP: ascorbyl palmitate; %EE: entrapment efficiency; PS: particle size; PDI: polydispersity index; and ZP: zeta potential.

**Table 4:** Analysis of variance (ANOVA) table for MFC-loaded ASPs optimization.

Responses	R^2^	Adjusted R^2^	Predicted R^2^	Adequate Precision	Significant Factors
%EE	0.970	0.962	0.955	32.64	X_1_, X_2_, X_3_
PS (nm)	0.983	0.962	0.800	21.48	X_1_, X_2_
PDI	0.986	0.976	0.942	26.52	X_3_
ZP (mV)	0.895	0.854	0.732	15.22	X_1_, X_3_

Abbreviations: MFC: Metformin hydrochloride; ASPs: aspasomes, %EE: entrapment efficiency percentage; PS: particle size; PDI: polydispersity index; and ZP: zeta potential.

**Table 5:** Measurement of mMASI score on both sides of the face before and after treatment.

	Right-Side OASP Formula	Left-Side Kligman’s Formula	*p* Value
Mean of mMASI before treatment, mean ± SD ^∞^	10.68 ± 2.68	10.67 ± 2.56	0.997
(Range)	(6.2–14.55)	(6.2–14.3)	
Mean of mMASI after treatment, mean ± SD ^∞^	4.17 ± 1.22	6.28 ± 1.41	0.002 *
(Range)	(2.6–5.8)	(5–9.1)	
P1 value (before vs. after treatment in each group) ^€^	<0.001 *	<0.001 *	
Reduction in mMASI after two months, mean ± SD ^∞^	6.51 ± 1.66	4.39 ± 1.73	0.012 *
(Range)	(3.6–8.75)	(1.2–7.65)	
Percent of reduction in mMASI after two months, mean ± SD ^∞^	61.09 ± 4.92	40.01 ± 10.02	<0.001 *
(Range)	(53.15–66.67)	(19.35–53.50)	

Data are presented as mean ± standard deviation (SD), (interquartile range [25th–75th percentile] (IQR)). Statistical analysis was conducted using ∞ independent-samples *t* tests and € paired-samples *t* tests. * *p*-values < 0.05 were considered significant. OASPs: optimal MFC-loaded aspasomes. mMASI: Modified Melasma Area Severity Index.

**Table 6:** Physician global assessment (PGA) of melasma after treatment on both sides of the face.

Clinical Response	Right-Side OASP Formula	Left-Side Kligman’s Formula	*p* Value
Excellent	6 (60%)	0 (0%)	0.001 *
Good	4 (40%)	7 (70%)	
Moderate	0 (0%)	3 (30%)	
Mild	0 (0%)	0 (0%)	

Data are presented as frequency and percentage. Statistical analysis was conducted using Chi-square test. * *p*-values < 0.05 were considered significant. OASPs: Optimal MFC-loaded aspasomes.

**Table 7:** Comparison of number of MART-1-positive-stained cells before and after treatment.

Parameter	Right Side of the Face OASP Formula	Left Side of the Face Kligman’s Formula	*p*-Value
Number of MART-1-positive cells in the epidermal layer before treatment, median (IQR) ^¥^	17 (10–22.8)	17.5 (9.8–21.5)	0.879
Number of MART-1-positive cells in the epidermal layer after treatment, median (IQR) ^¥^	5 (2.8–7.3)	7 (5.8–10.3)	0.044 *
Comparison of MART-1-positive cells in the epidermal layer before and after treatment in each group ^α^	0.005 *	0.005 *	
Number of MART-1-positive cells in the dermal layer before treatment, median (IQR) ^¥^	6.5 (3.8–9.3)	6.5 (4.5–10)	0.820
Number of MART-1-positive cells in the dermal layer after treatment, median (IQR) ^¥^	2.5 (0.8–4)	4.5 (2.8–5.3)	0.039 *
Comparison of MART-1-positive cells in the dermal layer before and after treatment in each group ^α^	0.005 *	0.005 *	
Total number of MART-1-positive cells before treatment, median (IQR) ^¥^	24.5 (18.3–28.5)	25.5 (17–28)	0.940
Total number of MART-1-positive cells after treatment, median (IQR) ^¥^	7 (4.5–10)	11 (8–16)	0.035 *
Comparison of the total number of MART-1-positive cells before and after treatment in each group ^α^	0.005 *	0.005 *	
Percent reduction in total MART-1-positive cells after treatment, median (IQR) ^¥^	63.64 (57.84–66.67)	36.61 (21.67–48.65)	0.0002 *

Data are presented as median and interquartile range (IQR) (25th–75th percentile). Statistical analysis was conducted using ^¥^ Mann–Whitney test, ^α^ Wilcoxon Signed rank test. * *p*-values < 0.05 were considered significant. MART-1: Melanoma antigen recognized by T-cells 1. OASP: Optimal MFC-loaded aspasome.

## Data Availability

The original contributions presented in this study are included in the article. Further inquiries can be directed to the corresponding author.

## Abbreviations

The following abbreviations are used in this manuscript:

%EE	Entrapment efficiency percent
PS	Particle size
PDI	Polydispersity index
ZP	Zeta potential
MFC	Metformin hydrochloride
AP	Ascorbyl palmitate
PC	Phospholipid
ASP	Aspasomes
